# Editorial: Football training and competition

**DOI:** 10.3389/fpsyg.2026.1922829

**Published:** 2026-08-05

**Authors:** Diogo Coutinho, Bruno Travassos, Rafael Ballester Lengua, Sigrid Olthof, Bruno Gonçalves

**Affiliations:** 1 Department of Physical Education and Sports Sciences, University of Maia (UMAIA), Maia, Portugal; 2 Research Center in Sports Sciences, Health Sciences and Human Development (CIDESD), Vila Real, Portugal; 3 Portuguese Football Federation, FPF Academy, Oeiras, Portugal; 4 Department of Sports Sciences, University of Beira Interior, Covilhã, Portugal; 5 Faculty of Physical Education and Sport Sciences, Catholic University of Valencia “San Vicente Mártir”, Torrent/Valencia, Spain; 6 Research Institute for Sport and Exercise and Sciences, Liverpool John Moores University, Liverpool, United Kingdom; 7 Universidade de Évora, Escola de Saúde e Desenvolvimento Humano, Departamento de Desporto e Saúde, Évora, Portugal; 8 Universidade de Évora, Comprehensive Health Research Centre (CHRC), Évora, Portugal

**Keywords:** artificial intelligence, competition, football, performance analysis, referee, small-sided games, soccer, talent development

Football (soccer) is the world's most popular sport, engaging millions of participants across recreational, semi-professional, and elite levels of play. This widespread appeal has long sustained a rich and expanding body of scientific inquiry. Yet the sport's continuous evolution, shaped by technological innovations, shifting training philosophies, advances in data analytics, and the growing professionalization of player development systems, demands that researchers and practitioners continually adapt their approaches. Against this backdrop, the Research Topic “*Football training and competition*” was conceived with a clear ambition: to stimulate scientific inquiry that bridges the gap between laboratory knowledge and the realities of the training pitch and competitive environment. Researchers were invited to submit original research, systematic reviews, perspectives, and commentaries across a broad spectrum of themes, from the design of training tasks and physical monitoring, to the use of artificial intelligence and the psychological dimensions of performance.

The response from the scientific community exceeded our expectations. Thirty-one articles, contributed by 133 authors representing institutions spanning Europe, Asia, South America, and Africa, were published across Frontiers in Psychology (Sport Psychology and Movement Science sections) and Frontiers in Sports and Active Living. In this editorial, we synthesize these contributions across five thematic domains: (i) training design and optimization; (ii) physical monitoring and load management; (iii) perceptual-cognitive skills and decision-making; (iv) competition analysis and artificial intelligence; and (v) psychological, psychosocial, and behavioral dimensions, including referee-related topics.

## Training design and optimization

A central theme of this Research Topic was the design of effective training environments that maximize players' physical, technical, and tactical development. Several articles addressed how structural task manipulations, particularly in small-sided games (SSGs), and pedagogical approaches shape learning outcomes. Rumpf et al. provided a comprehensive literature review and practical guide on the effects of relative pitch size on physiological, physical, technical, and tactical variables in SSGs, synthesizing an extensive body of evidence and offering practical guidance for coaches seeking to tailor SSG configurations to specific training objectives. Extending this theme to youth populations, Bleidelis et al. compared box-to-box and positional possession game formats in U13 footballers, offering initial evidence that game format meaningfully shapes the technical and physical responses of young players, with implications for age-appropriate training design.

Beyond the structural features of training tasks, the pedagogical approach adopted by coaches is equally consequential. Yang et al. investigated the differential effects of linear and non-linear pedagogy on motor skill performance, revealing that individual adaptability moderates the relationship between pedagogical strategy and outcomes: players with higher adaptability derived greater benefit from non-linear approaches. This finding reinforces the growing consensus that effective coaching requires individualized, constraints-based strategies rather than one-size-fits-all approaches. Complementarily, Coutinho et al. examined the role of priming videos, specifically, footage emphasizing offensive creativity, in shaping youth players' behavior during SSGs. Exposure to creative and offensive priming content prior to practice enhanced players' attacking behavior and creative expression on the pitch, suggesting that video-based priming represents an accessible and effective pedagogical tool for coaches aiming to foster players' offensive intent.

Together, these contributions suggest that training design should be understood not only as the manipulation of task formats, but also as the creation of learning environments that interact with players' individual constraints. The studies on non-linear pedagogy and video priming illustrate how adaptability and prior informational cues may shape players' opportunities for action and creative behavior during small-sided games.

## Physical monitoring and load management

Understanding the physical demands of football, across training and competition, is fundamental to optimizing performance and managing player health. Ribeiro et al. examined training exercises, match demands, and variability in elite futsal, identifying meaningful discrepancies between the stimuli encountered in training and those experienced during competition. These findings carry practical implications for how practitioners design preparation programs and select training tasks that adequately replicate competitive demands. The question of how to contextualize and normalize external load metrics was addressed in a perspective article by Pimenta et al. who argued for the relativization of high-speed running (HSR) data to account for individual differences in maximum sprint speed. Their theoretical-practical discussion highlights how raw GPS metrics may misrepresent actual physiological strain unless placed within an individual frame of reference, an important methodological consideration for sports scientists working in applied settings. On the topic of peak demands, Yousefian et al. characterized multifactorial peak kinematic and mechanical intensities in elite youth football, illustrating the complexity of concurrent physical stressors and underscoring the need for multidimensional monitoring approaches that go beyond single-variable analyses.

Neuromuscular monitoring was addressed in multiple contributions. Ferrari et al. documented changes in lower-limb neuromuscular performance from the pre-season to the early competitive period in elite male professional football players, identifying a trajectory of neuromuscular adaptation that can inform periodization decisions. Lorigados Pérez and Bustamante-Sánchez conducted a systematic review evaluating tensiomyography (TMG) as a monitoring tool for the knee flexor and extensor musculature in elite football players. Their synthesis confirms TMG's utility for tracking individual neuromuscular state, functional fatigue, and lateral asymmetries throughout the competitive season, offering practical guidance for medical and coaching staff. Extending the assessment toolkit for youth players at a more applied level, Schulz et al. examined the utility of handgrip strength (HGS) as a practical performance indicator in 221 elite male youth footballers aged 11–19 years. HGS was strongly associated with jumping and sprinting performance, with age and biological maturation moderating these relationships, suggesting that this simple, low-cost measure holds real potential for talent monitoring and performance prediction when individual developmental stage is taken into account.

The potential of wearable technology to enrich training monitoring was demonstrated by Kranzinger et al. who tracked performance development in female youth football players over a 14-month period using foot-mounted inertial measurement units (IMUs). By relating objective high-percentile performance metrics (ball speed, peak speed, distance) to players' subjective post-training ratings of intensity and happiness, the authors revealed meaningful associations that support the integration of both subjective and objective data within holistic player monitoring pipelines.

Taken together, these contributions reinforce the need to move from isolated metrics toward individualized and multidimensional monitoring methods. Contextualized actions and corresponding demands are key to further understanding the game The integration of information related with biological maturation, and subjective responses all provide complementary information for interpreting players' training and competition demands.

## Perception and action

Football performance is deeply intertwined with players' ability to perceive, anticipate, and respond to dynamic environmental information. Several contributions examined how these skills can be assessed, trained, and enhanced. Libreau and Benguigui showed that a video training intervention focusing on ball trajectory anticipation improved women's team performance in corner kick situations, demonstrating that targeted perceptual training can produce transferable gains in game-relevant contexts. Complementing this, Ivosevic and Stöckel demonstrated that skilled football players exploit early visual cues for pattern recognition in corner kick scenarios, suggesting that anticipatory advantages in experts arise from sensitivity to structural regularities in opponent behavior that novices do not readily perceive.

The role of visual exploration in football was addressed through a training intervention study by Hintermann et al. whose 5-week “Heads Up Girls!” program systematically improved scanning behavior in U19 female players from both elite and grassroots teams. The improvements observed across contexts suggest that scanning can be meaningfully trained and that targeted interventions have practical relevance beyond elite settings. At the frontier of applied neuroscience, Wang et al. investigated the effects of transcranial direct current stimulation (tDCS) on neuromuscular control strategies during penalty kicks. Their findings provide early evidence that non-invasive brain stimulation can modulate the neural underpinnings of specific football skills, opening a promising avenue for interdisciplinary research into brain-body interactions in high-precision motor tasks.

Together, these studies map a continuum of perceptual-cognitive enhancement, ranging from foundational visual exploration and pattern recognition to acute neurofunctional modulation. These insights collectively emphasize that modern training in football needs to combine ecological training with emerging neuroscientific approaches, while future work should further examine transfer to competitive decision-making and skilled action.

## Performance analysis

The analysis of football performance across multiple competitions and levels of play was well-represented in this Research Topic. Liu et al. explored the factors influencing foul frequency in football, identifying team rating, game state, and match phase as key determinants; findings with direct implications for referee preparation and tactical understanding of competitive dynamics. At the tournament level, Martín-Castellanos et al. analyzed key metrics distinguishing advancing from eliminated teams at the male UEFA EURO 2024, highlighting goals, shots on target, pressing indicators, and possession-related variables as significant discriminators. The question of how substitutions affect match outcomes was examined by Gabrys et al. who studied the timing and type of substitutions made by national team coaches at EURO 2024, providing data-driven insights into the tactical decision-making processes of coaches operating under high-stakes tournament conditions.

Team performance dynamics beyond individual matches were explored by Campos et al. who examined the impact of international call-ups on clubs' offensive and defensive performance indicators. Their results suggest that squads with more players summoned to national teams exhibit changes in clubs' collective performance profiles, including possession-related and attacking indicators, carrying practical implications for squad management and competitive calendar planning. The use of network science to understand team performance and tactical behavior was synthesized by Alves et al. in a systematic review of social network analysis (SNA) in football, mapping the current state of knowledge and identifying meaningful methodological and applied gaps that future research should address.

Forcher et al. directly addressed the use of artificial intelligence in football, comparing expected possession value (EPV) and expected goals (xG) as predictors of match outcomes in the Bundesliga. EPV, by capturing the value of possession actions, EPV offers a complementary perspective to xG, although the relative predictive value of both metrics depends on the analytical scenario considered. This contribution, alongside the machine learning pipeline applied by Kranzinger et al. in tracking female youth football performance, reflects a growing trend: the integration of AI-derived metrics and data-driven pipelines is reshaping both the practice and science of football analysis.

This body of work highlights an ongoing shift within performance analysis from descriptive performance indicators toward more integrated modeling of collective behavior. Tournament-level metrics, network science, and advanced metrics such as EPV can enrich the interpretation of match dynamics, although their applied value depends on contextual validation, model interpretability, and translation into actionable information for coaches and practitioners.

## Psychological, psychosocial, and behavioral dimensions

Consistent with the holistic aims of this Research Topic, a substantial cluster of articles addressed the psychological, emotional, social, and behavioral dimensions of football performance. These contributions can be organized into four complementary areas: coach and player psychological functioning, youth social-developmental experiences, refereeing, and female player health.

At the coaching level, Rausch et al. conducted a qualitative study on football coaches' strategies during performance crises, identifying communication, player rotation, and individualized feedback as central mechanisms through which coaches attempt to manage periods of poor form. These insights have direct relevance for coaching education curricula and for the support structures that clubs provide to their coaching staff. At the player level, Şekerci et al. examined the interplay of emotional intelligence and mental toughness in amateur football, finding that goal commitment mediates the relationship between the two constructs. Pekel et al. investigated resilience in professional football players, demonstrating that humor acts as a buffering mechanism between occupational strain and resilience, encouraging practitioners to consider psychological flexibility alongside traditional mental skills training. Non-verbal behavior on the pitch was the focus of Lian et al., who analyzed football players' body language at the FIFA men's World Cup 2022, revealing systematic variation in non-verbal cues across different match phases and situational contexts. This underexplored dimension of player behavior provides new entry points for understanding confidence, team cohesion, and communication under competitive pressure.

Social and developmental dimensions of football participation were addressed in two articles. Wu et al. identified distinct friendship quality profiles among Chinese adolescent football players using latent profile analysis, revealing meaningful variation in the social experience of participation that may influence long-term engagement and wellbeing. In youth development settings, Hanstad et al. conducted a longitudinal case study on the balance between deliberate play and deliberate practice in grassroots football, finding that coaches dynamically adjusted their emphasis based on developmental context, nuancing the prevailing debate on optimal practice structures for young footballers and highlighting the importance of practitioner responsiveness in talent development environments.

Referee science, a historically underrepresented domain in football research, received dedicated attention in this Research Topic. Taylor et al. examined the role of organizational culture and talent development pathways in football referee career progression, highlighting systemic factors beyond individual technical competence that shape referees' trajectories. The impact of the Video Assistant Referee (VAR) system was investigated from two geographic perspectives. Johansen et al. analyzed disciplinary decision-making in the English Premier League before and after VAR implementation, showing changes in disciplinary outcomes and suggesting that VAR may support the identification of clear and obvious errors. Kubayi extended this analysis to the Africa Cup of Nations, examining VAR's context-dependent effects on gameplay and refereeing-related indicators, and contributing evidence from African football contexts that are frequently absent from the mainstream literature.

Finally, this Research Topic included an important contribution addressing female football specifically. Santos et al. investigated the impact of the menstrual cycle and associated barriers on the performance of Portuguese football and futsal players, bringing to light health management challenges that remain insufficiently addressed in research and practice. This work is a timely reminder that female athletes require dedicated scientific attention and evidence-based support structures tailored to their specific physiological and contextual needs.

This diverse cluster highlights the necessity of viewing football performance as a multi-layered ecosystem. By linking individual psychological traits and social dynamics with broader systemic factors, these contributions underscore the central role of the human element in performance and development. Optimizing competitive and developmental environments therefore requires addressing psychosocial and contextual realities alongside traditional physical and tactical dimensions.

## Conclusions and future directions

**Figure 1:**
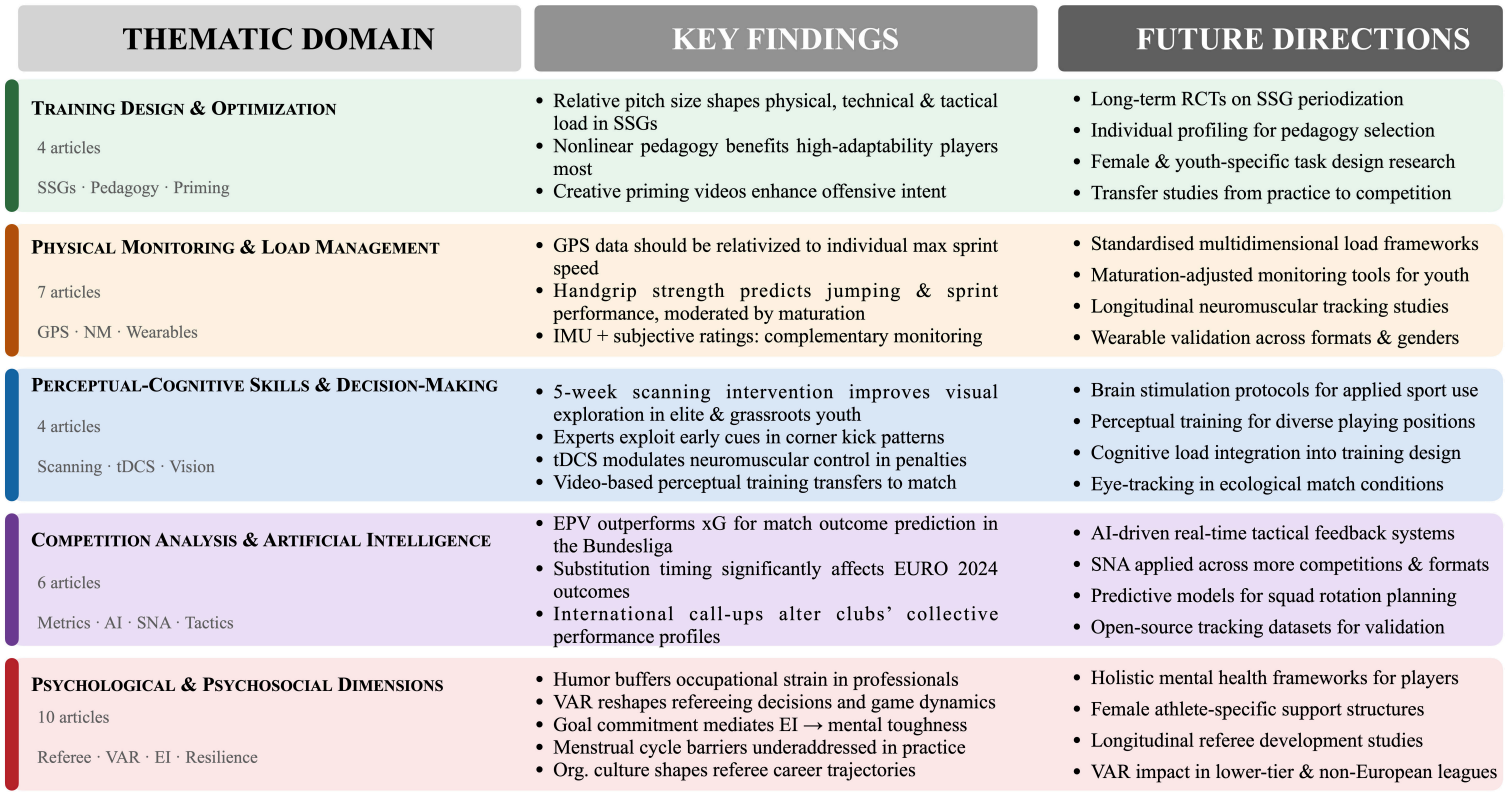
Key findings and future research directions. NM, Neuromuscular; SSG, Small-Sided Game; EI, Emotional Intelligence; EPV, Expected Possession Value; SNA, Social Network Analysis; VAR, Video Assistant Referee.

Collectively, the 31 articles comprising this Research Topic reflect the remarkable breadth and depth of contemporary football science (see Figure 1). The contributions span a wide range of designs, from randomized training interventions and longitudinal monitoring studies to systematic reviews, machine learning applications, and qualitative inquiry, and include populations ranging from grassroots youth players to elite professionals, male and female, across multiple football formats including football and futsal.

Several cross-cutting themes emerge from this body of work. First, the integration of technology, wearables, artificial intelligence, VAR, and non-invasive brain stimulation is reshaping both the practice of football and the science that underpins it. Researchers and practitioners would benefit from sustained collaboration to ensure that technological innovations are evaluated rigorously and implemented responsibly. For technology-based approaches, future studies should prioritize external validation, model interpretability, and open datasets that allow independent replication.

Second, a more holistic and multidimensional approach to player development and performance analysis is increasingly recognized as necessary. Contributions to this Research Topic consistently highlight the limitations of siloed, single-dimension analyses and point toward integrated frameworks that account for the physical, technical, tactical, cognitive, emotional, and social dimensions of performance simultaneously. Third, while this Research Topic made meaningful contributions to female football and youth football research, these populations remain underrepresented. The field would benefit substantially from a more deliberate and sustained effort to recruit these populations, as findings from elite male adult football cannot be assumed to generalize. In youth football, future research should pay particular attention to biological maturation, relative age, and individual developmental timing, as these factors shape how training load, performance indicators, and talent-related decisions are interpreted.

Fourth, applied contexts, particularly the design of ecologically valid training environments and the translation of research findings to coaching practice, deserve continued emphasis. As club structures become increasingly open to collaboration with researchers, the conditions for impactful applied research are more favorable than ever. We hope this Research Topic serves as a valuable and enduring resource for researchers, coaches, practitioners, and all those committed to advancing the science and practice of football.

Across domains, the Research Topic reinforces the importance of considering players' individuality and developmental trajectories when contextualizing practice and performance, avoiding general conclusions. Also, from the overall studies it was stressed the need for research designs that are more longitudinal, consider chronic instead of acute adaptations, and providing greater ecological validity.

